# High-Quality Transmission of Cardiotocogram and Fetal Information Using a 5G System: Pilot Experiment

**DOI:** 10.2196/19744

**Published:** 2020-09-08

**Authors:** Katsuhiko Naruse, Tomoya Yamashita, Yukari Onishi, Yuhi Niitaka, Fumikage Uchida, Kazuya Kawahata, Mayu Ishihara, Hiroshi Kobayashi

**Affiliations:** 1 Department of Obstetrics and Gynecology Nara Medical University Kashihara, Nara Japan; 2 NTT DoCoMo, Inc Tokyo Japan; 3 TOITU Co, Ltd Tokyo Japan

**Keywords:** 5G communication system, home telecare, online health, telecardiology, telemedicine, teleconsulting

## Abstract

**Background:**

A cardiotocogram (CTG) is a device used to perceive the status of a fetus in utero in real time. There are a few reports of its use at home or during emergency transport.

**Objective:**

The aim of this study was to test whether CTG and other perinatal information can be transmitted accurately using an experimental station with a 5G transmission system.

**Methods:**

In the research institute, real-time fetal heart rate waveform data from the CTG device, high-definition video ultrasound images of the fetus, and high-definition video taken with a video camera on a single line were transmitted by 5G radio waves from the transmitting station to the receiving station.

**Results:**

All data were proven to be transmitted with a minimum delay of less than 1 second. The CTG waveform image quality was not inferior, and there was no interruption in transmission. Images of the transmitted ultrasound examination and video movie were fine and smooth.

**Conclusions:**

CTG and other information about the fetuses and pregnant women were successfully transmitted by a 5G system. This finding will lead to prompt and accurate medical treatment and improve the prognosis of newborns.

## Introduction

A cardiotocogram (CTG) is a device used worldwide in obstetrics to perceive the status of a fetus in the second half of the gestational period in utero in real time. It has been relied upon as a test comparable to fetal ultrasound because a decelerated heart rate detected by this method indicates fatal stress in the fetus, an accelerated heart rate baseline indicates infection in the fetus in utero, and a fair baseline variability indicates the sparing ability of the fetus. However, at present, it is used only in medical care facilities, and there are few reports of its use at home or during emergency transport. In addition, there are many limitations to the prehospital diagnosis that can be made during transport of perinatal diseases [1], and there is an urgent need to develop a device that helps paramedics to recognize obstetric abnormalities.

Recently, wireless/mobile transmission using the next generation of high-speed, high-capacity systems, so-called 5G [2], has been developed in some parts of the world, and it is expected to be applied to the medical field, mainly because of its high speed and good compatibility with cloud computing [3]. It can also be used as a tool for accurately conveying data to medical institutions at home or during patient transfers [3]. However, no research has been conducted yet in the field of obstetrics to verify its utility.

In this pilot study, we tested whether CTG and other perinatal information can be transmitted accurately using an experimental station with a 5G transmission system.

## Methods

This study was conducted in December 2019 at the Research Institute of NTT DoCoMo Incorporated (Osaka, Japan). We combined real-time fetal heart rate waveform data of 30 minutes from the MT-610 CTG device (TOITU Co, Ltd), which stores and reproduces anonymized data from actual normal fetuses; high-definition (HD) video ultrasound images of the fetus (Prosound α10, Hitachi Ltd; prerecorded and anonymized, repeat of the 10-minute scan); and HD video taken with a common digital video camera on a single 5G line. All data were transmitted by 4.5 GHz radio waves (selected from the radio wave bands 3.7 GHz, 4.5 GHz, and 28 GHz available for 5G in Japan) from the transmitting station to the receiving station, which is housed at the institute on the same floor of the building. All of this information was reproduced on a computer on the receiving end that simulated a secondary hospital. The CTG waveforms and ultrasound movie images that could be seen on the receiving screen were checked by an obstetrics and gynecology consultant (KN) to confirm if they were acceptable for diagnosis. Another consultant (HK) later checked them again from the stored images. The face of a female model was featured in the HD video, and the complexion and expression of the model could be recognized after the transmission. The model also held a digital clock, so that the delay in data transmission could be defined later.

## Results

**Figure 1:**
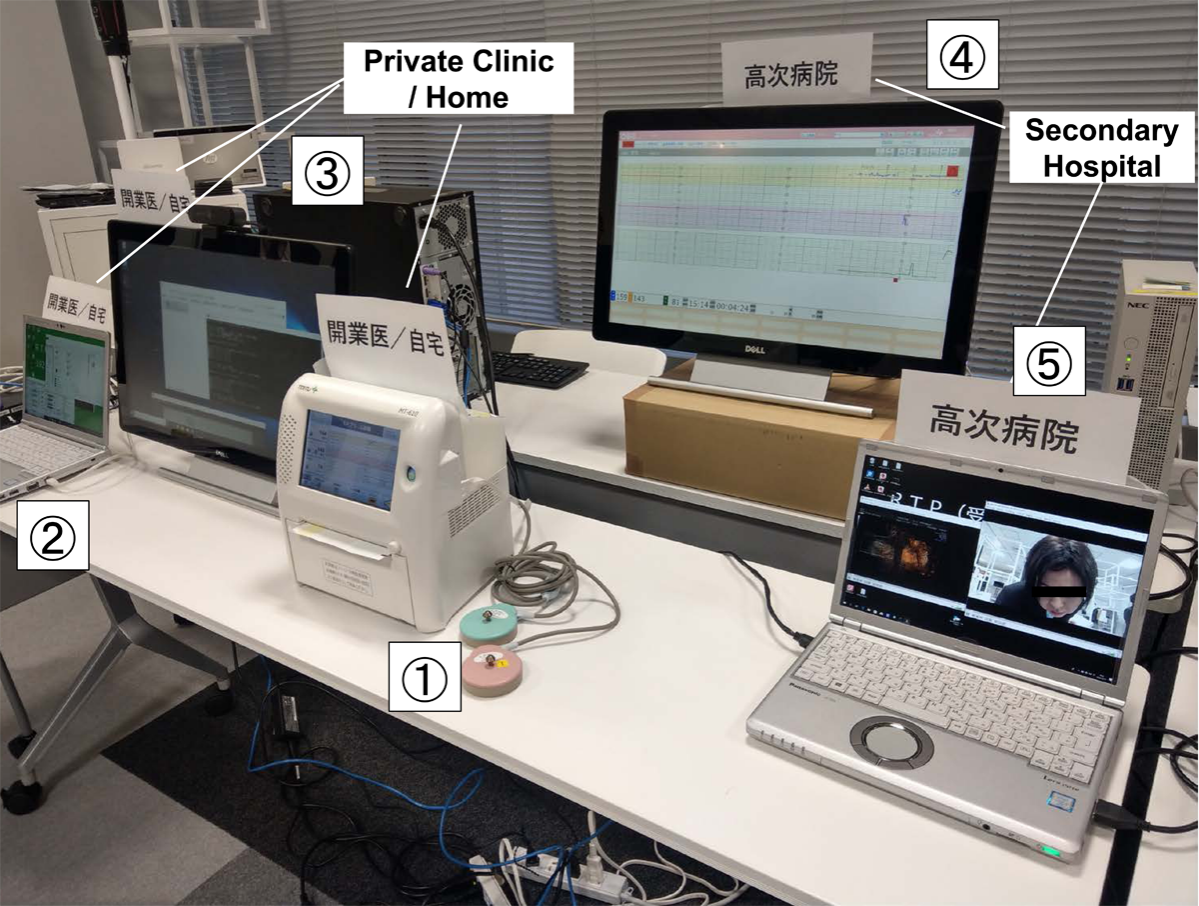
Panoramic view of the experiment. Objects 1-3 are the source side, and objects 4 and 5 are the receiving side of the 5G transmission system. 1: The cardiotocogram (CTG) device (MT-610) transmits the waveform of the fetal heart rate in clinical practice; 2: the computer plays back ultrasound examination videos; 3: the computer plays back movies from a video camera (operation of the real-time self-taken video is checked afterwards); 4: the CTG waveform display is maximized at the receiving end to check visibility; 5: movies of the ultrasound examination (left) and video camera (right) are played simultaneously at the receiving end.

Connection of the devices using the 5G transmission system from the source side (1, 2, and 3 in Figure 1) and receiver side (4 and 5 in Figure 1) via a radio wave transmitter and receiver on the same floor was successful.

All data were proven to be transmitted with a minimum delay of less than 1 second as per the digital clock in the HD video. The CTG waveform image quality was not inferior, and there was no interruption in transmission. The fetal heart rate was clearly legible, and the variability of the baseline was easy to read. At the same time, the real-time images of the transmitted ultrasound examination were fine and smooth, and no unnatural movements were observed. In addition, the color of the model’s face and surrounding movements in the video image were correctly transmitted. There were no unnatural color tones; as a result, we were able to recognize her complexion and expression (Figure 1).

## Discussion

To our knowledge, this study is the first to show that CTG and other information about fetuses and pregnant women can be transmitted by a 5G system. A system to monitor CTGs within a medical care facility via an intranet is common, and a system to exchange CTG waveforms between medical institutions via the internet has been put to practical use. However, attempts to send data from a pregnant woman's home to a medical institution via a mobile network or to send data from an ambulance or air ambulance in transit have not been sufficiently carried out, except in one case in 1992 [4]. If fetal information and other data can be transmitted via a mobile network, medical institutions will be able to keep abreast of sudden changes in the condition of the fetus at home or of pregnant women with complications being transported to the hospital, which will lead to prompt and accurate medical treatment and improve the prognosis of the newborn.

5G systems are anticipated to have the potential to significantly improve the speed and stability of transmission in mobile communications. In this study, we found that not only CTG, but also images during ultrasound examinations and high-quality videos of people who mimicked patients could be transmitted without problems. Although our study was performed in a laboratory, and the distance between the transmitter and receiver was not far, recent planning of information technology infrastructure on 5G transmission promises better linkage of devices under both low or high power [5]. In the field of emergency medicine, doctors and paramedics have already started attempting to make accurate diagnoses by using smartphones to take videos, through which consultants at a central hospital can assess the situation in real time [6]. 5G systems are expected to be able to transmit high-quality CTGs as well as more precise ultrasound images of the fetus and HD videos reporting the complexion, expression, behavior, and complaints of pregnant women/patients and the surrounding environment. Developing new solutions for the new era of 5G systems for the home care of pregnant women and emergency transport systems in perinatal care may not only dramatically improve the prognosis for mother and child, but also reduce the burden on health care providers.

Additionally, as predicted by Oleshchuk and Fensli [7], the 5G system will also work with artificial intelligence and big data to enable “self-determined medicine” to make instant diagnoses based on data obtained at home [3]. Issues such as developing new infrastructure for 5G transmission, shorter propagated distance of the radio wave on higher frequency, or data privacy (problems not only for 5G) remain to be solved. However, home monitoring of a fetus with the 5G system is a particularly good application of this new generation of technology, which could create a new future for obstetric care.

## Abbreviations

CTG: cardiotocogram
HD: high definition
